# Hockey Fans in Training (Hockey FIT) pilot study protocol: a gender-sensitized weight loss and healthy lifestyle program for overweight and obese male hockey fans

**DOI:** 10.1186/s12889-016-3730-5

**Published:** 2016-10-19

**Authors:** Dawn P. Gill, Wendy Blunt, Ashleigh De Cruz, Brendan Riggin, Kate Hunt, Guangyong Zou, Shannon Sibbald, Karen Danylchuk, Merrick Zwarenstein, Cindy M. Gray, Sally Wyke, Christopher Bunn, Robert J. Petrella

**Affiliations:** 1 Centre for Studies in Family Medicine, Department of Family Medicine, Schulich School of Medicine and Dentistry, Western University, London, ON Canada; 2 School of Health Studies, Faculty of Health Sciences, Western University, London, Canada; 3 School of Kinesiology, Faculty of Health Sciences, Western University, London, Canada; 4 MRC/CSO Social and Public Health Sciences Unit, Institute of Health and Wellbeing, College of Medical, Veterinary and Life Sciences, University of Glasgow, Glasgow, UK; 5 Department of Epidemiology and Biostatistics, Schulich School of Medicine and Dentistry, Western University, London, Canada; 6 Robarts Clinical Trials, Robarts Research Institute, Western University, London, Canada; 7 The Schulich Interfaculty Program in Public Health, Schulich School of Medicine and Dentistry, Western University, London, Canada; 8 Institute of Health and Wellbeing, College of Social Sciences, University of Glasgow, Glasgow, UK

**Keywords:** Overweight/Obese men, Lifestyle intervention, Health promotion, Hockey, Sport fan, Masculinity, Health technology, Weight loss, Physical activity, Healthy eating

## Abstract

**Background:**

Effective approaches that engage men in weight loss and lifestyle change are important because of worldwide increases, including in Canada, in obesity and chronic diseases. Football Fans in Training (FFIT), developed in Scotland, successfully tackled these problems by engaging overweight/obese male football fans in sustained weight loss and positive health behaviours, through program deliveries at professional football stadia.

**Methods:**

Aims: 1) Adapt FFIT to hockey within the Canadian context and integrate with Health*e*Steps™ (evidence-based lifestyle program) to develop Hockey Fans in Training (Hockey FIT); 2) Explore potential for Hockey FIT to help overweight/obese men lose weight and improve other outcomes by 12 weeks, and retain these improvements to 12 months; 3) Evaluate feasibility of recruiting and retaining overweight/obese men; 4) Evaluate acceptability of Hockey FIT; and 5) Conduct program optimization via a process evaluation. We conducted a two-arm pilot pragmatic randomized controlled trial (pRCT) whereby 80 overweight/obese male hockey fans (35–65 years; body-mass index ≥28 kg/m^2^) were recruited through their connection to two junior A hockey teams (London and Sarnia, ON) and randomized to Intervention (Hockey FIT) or Comparator (Wait-List Control). Hockey FIT includes a 12-week Active Phase (classroom instruction and exercise sessions delivered weekly by trained coaches) and a 40-week Maintenance Phase. Data collected at baseline and 12 weeks (both groups), and 12 months (Intervention only), will inform evaluation of the potential of Hockey FIT to help men lose weight and improve other health outcomes. Feasibility and acceptability will be assessed using data from self-reports at screening and baseline, program fidelity (program observations and coach reflections), participant focus group discussions, coach interviews, as well as program questionnaires and interviews with participants. This information will be analyzed to inform program optimization.

**Discussion:**

Hockey FIT is a gender-sensitive program designed to engage overweight/obese male hockey fans to improve physical activity and healthy eating choices, thereby leading to weight loss and other positive changes in health outcomes. We expect this study to provide evidence for a full-scale confirmatory pRCT.

**Trial registration:**

NCT02396524 (Clinicaltrials.gov). Date of registration: Feb 26, 2015.

## Background

A significant proportion of men of all ages are overweight or obese. In 2014, 21.8 % of Canadian men self-reported height and weight that classified them as obese (i.e., using body mass index, BMI) and 40.0 % of Canadian men were classified as overweight [1]. A recent study by the Global BMI Mortality Collaboration, analyzing records of 3.95 million people from 189 studies worldwide, showed that men had higher all-cause mortality for every additional five BMI points over 25 kg/m^2^ (relative to BMI 20–25 kg/m^2^), compared to women (hazard ratios and 95 % CIs: 1.51 (1.46–1.56) for men vs 1.30 (1.6–1.33) for women) [2]. The increased risk of death was also higher in younger people [2].

Men suffer poorer health outcomes on many measures of health status compared to women [3–5]. Men seem less knowledgeable about health risk factors [6]; and are less likely to access, interpret and apply information to maintain and improve their health [7]. According to the 2012–2013 Canadian Health Measures Survey, only 24 % of men achieved the recommended levels of physical activity, and the majority spent their waking hours being sedentary [8]. Despite this, the issue of gender is often neglected when planning and implementing health promotion and chronic disease prevention and management strategies.

Chronic diseases share several lifestyle risk factors including obesity/overweight status, physical inactivity, sedentary lifestyle, unhealthy eating habits and low health literacy, which if reduced, could prevent or eliminate most chronic diseases and their complications [9]. In Canada, these trends [1], which are more pronounced in rural and small urban settings, may help explain the higher prevalence of type 2 diabetes mellitus and increased mortality rates found in these regions [10]. Since 2003, there has been a steady increase in the number of men who are obese in Canada; in 2003, 16 % of men were obese compared to 21.8 % in 2014 [1]. These trends are alarming and pose threats to men’s individual health and the health care system as a whole.

Men may deny illness or delay treatment, ignore health concerns or engage in high-risk behaviours to prove adherence to the construct of masculine toughness or strength [3, 11–14]. Men’s behavioural choices are also influenced by culture, class, ethnicity, context, age and life events [3]. Even so, increasing evidence shows that when gender issues are used to inform program design and delivery, men will engage in weight management initiatives [12, 15–17]. Men may also be more open to participating in physical activity that is linked in some way to sport, athleticism, and competition [18] Although this could exclude certain settings or ages, there is evidence that walking – a universally accessible form of physical activity – is appealing, at least when introduced within the right context [19].

While health promotion programs targeting men have been implemented in other countries including Australia and the United Kingdom [20], programs have only recently started to emerge in Canada [21], and few options exist, particularly in rural and small urban regions of Canada where the burden of chronic disease is greater [10]. In general, men are reluctant to engage in healthy lifestyle and weight loss programs, seem less aware than women of their overweight status [22, 23], and associate increased body size with muscularity and masculinity [24, 25].

Physicians can play an important role in the dissemination of physical activity recommendations [26], as approximately 82 % of Canadians have reported having a regular doctor place they go for care [27], Most physicians, however, do not regularly assess or prescribe physical activity as part of a routine care, and even when discussed, few provide specific recommendations [28].

In a survey conducted in men living in small urban and rural communities in Canada, we found the following healthy lifestyle strategies appealed to men: 1) physical activity goals set with the help of a coach; 2) training for an event such a charity walk or sport; 3) in-person sessions with a coach and their peers in a fun atmosphere; and 4) technology-supported healthy lifestyle tools. A common theme that arose from our survey was the importance of support from other men. Men also frequently indicated that they would be motivated to lead a healthier lifestyle if it made them feel better (e.g., more energy, less pain, better sleep) and improved their fitness and memory [29, 30].

Men appear to harbour misperceptions about the dietary behaviours required to lose weight [23, 31, 32] and perceive dieting and organized weight loss programs as ‘feminized’ domains [33]. Hence, interventions that target men will be more likely to succeed if they encompass physical activity as well as diet [34, 35] and similar to our findings, if they include peer groups they can identify with [12, 16, 36]. Programs situated in a sporting context where participants had a strong sense of affiliation have been found to be both effective and to have low dropout rates, due to high ratings of participant satisfaction [7, 17].

The social and psychological connections (e.g., identity, validation, belonging) that being a fan creates are powerful [37], and this is particularly true in group contexts [36]. Using a pragmatic Randomized Controlled Trial (pRCT) design, Football Fans in Training (FFIT), a gender-sensitised, weight loss and healthy living program developed for delivery to men through Scottish professional football clubs by club coaches [15], demonstrated that the professional football club setting was a powerful ‘draw’ for overweight and obese men at high risk of future ill-health across the socio-economic spectrum and clinically significant weight loss and cost-effectiveness 12 months after baseline measures [38].

Canadian hockey is core to Canadian culture: two-thirds of adult Canadians follow the game as a fan and 8 out of 10 identify hockey as a key part of what it means to be Canadian [39]. In many areas, particularly small towns and rural areas, junior hockey clubs are the primary fan-base. These junior hockey clubs are often a rallying point in these communities, attracting a large number of fans (disproportionately male) to games, often more than in urban areas. The Ontario Hockey League (OHL), a semi-professional Junior A hockey league, is a member of the Canadian Hockey League, with one of the world’s largest hockey fan-bases; for example, last season, more than 9 million fans attended Canadian Hockey League games, many in small urban centres [40].

Hockey Fans in Training (Hockey FIT) may provide a preferred sport-fan venue to engage at-risk Canadian men. Hockey FIT will be based on FFIT, but adapted to hockey within the Canadian context, and, to strengthen sustainability, will be integrated with components of our lifestyle prescription program (Health*e*Steps™). The Health*e*Steps™ program is grounded in scientific evidence [41–46] and members of our pan-Canadian Health*e*Steps™ Network have demonstrated that the program can be applied to numerous community-based settings in urban and rural areas.

## Methods

### Study aims

1. Adapt FFIT to hockey within the Canadian context, and then integrate this with Health*e*Steps™ to develop Hockey FIT for delivery in Ontario Junior A Hockey Team sites (within the OHL);
2. Explore the potential for Hockey FIT to help overweight or obese men lose weight, increase their physical activity levels, and make improvements in other lifestyle and health-related outcomes by 12 weeks, and then retain these improvements to 12 months;
3. Evaluate feasibility of: a) recruiting men who are overweight or obese into a program focused on physical activity, exercise and healthy eating delivered in collaboration with OHL teams by trained Hockey FIT coaches); and b) retaining these men in both the program and for measurement sessions;
4. Evaluate the acceptability of the Hockey FIT program and research procedures;
5. Conduct Hockey FIT program optimization via a process evaluation.

### Aim 1: Adaptation and integration of FFIT and Health*e*Steps™ to create Hockey FIT

Hockey FIT was adapted from FFIT for the Canadian context using the love of hockey to encourage middle-aged, overweight and obese men to lose weight, increase physical activity and live a healthier lifestyle.

The Principal Investigator and other members of the Central Research Team (including the coach for the London site) went to Glasgow, Scotland in January 2015 to meet with FFIT researchers and observe FFIT sessions. While there, research team members attended two 90-min FFIT sessions at different clubs and were introduced to the format used to deliver FFIT programs, and coaching techniques to make the sessions enjoyable and interactive. The FFIT Coach Handbook was used as a reference for creating Hockey FIT sessions and developing the Hockey FIT Coach and Participant Handbooks.

We adopted the study design used by FFIT (see Methods section), but instead of targeting at-risk male soccer fans as was done in Scotland, we focused on hockey fans in Canada. The lifestyle prescription and the technology support tools protocols from the Health*e*Steps™ program [43, 47] were incorporated into the adapted FFIT approach, to develop Hockey FIT. As part of the Health*e*Steps™ program, participants receive individualized healthy living prescriptions for exercise, physical activity (step counts) and healthy eating, and are supported in-person to achieve their prescriptions and set goals with the help of a trained coach. To support longer-term maintenance of healthy lifestyles, participants are also offered free-of-charge access to technology-based coaching and support options (e.g., smartphone apps, private web-based social network, and telephone coaching).

Throughout the design of Hockey FIT, the research team made changes to the FFIT program design, to ensure the content followed Canadian guidelines and would resonate with Canadians. This included changing wording and doing conversions in some of the measurement tools (e.g., chips to fries; converting units of pints to cups; etc.). As well, Canadian resources were substituted in place of their British counterparts (e.g., Canada’s Food Guide [48] and Canadian Physical Activity Guidelines [49]). During FFIT, all sessions took place at the home stadium of the soccer team and were run by club community coaches (i.e., employees of the soccer clubs). For Hockey FIT, sessions were held at either the home arena of the hockey team or at a local fitness club and coaches were University-level students.

The Coach and Participant Handbooks were edited and reviewed by the Hockey FIT coaches, the Principal Investigator and other research team members in development of the final versions. The coaches ran five mock Hockey FIT sessions, using members of the research team and their friends and/or family as study participants. The research team also met weekly during the Hockey FIT program delivery to discuss progress, fidelity and further changes to the program.

### Aim 2: Potential for Hockey FIT to positively impact health outcomes

#### Study design

**Fig. 1:**
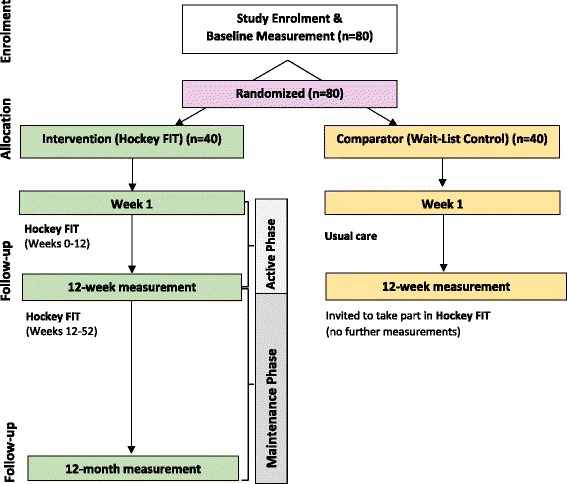
Flow Diagram for the Hockey Fans in Training Pilot Pragmatic Randomized Controlled Trial

We conducted a 12-week, two-arm, pilot pRCT of Hockey FIT, as well as a process evaluation of the Hockey FIT program, in which male hockey fans of two OHL teams (London Knights and Sarnia Sting), were individually randomized (1:1) to the Intervention (Hockey FIT) group (starting Hockey FIT program immediately) or the Comparator (Wait-List Control) group (starting Hockey FIT program after a 12-week delay). The 12-week active phase of the Hockey FIT intervention was followed by a 40-week maintenance phase. Measurement sessions were conducted at baseline and 12 weeks (both groups) and then again at 12 months (intervention group only). See Fig. 1 for study flow diagram.

Study recruitment began in April 2015 and the intervention periods started on May 12, 2015 (London site) and May 27, 2015 (Sarnia site). Western University Health Sciences Research Ethics Board approved this study and all participants provided written informed consent. The pRCT portion of this study was registered on February 26, 2015 with ClinicalTrials.gov (identifier: NCT02396524).

#### Location

The Central Research Team is located at Western University (London, ON, Canada). The London Knights team is in a medium-sized media market in London, Ontario (population 366,151 in 2011), and the Sarnia Sting team is in a smaller urban centre in Sarnia, Ontario (population 89,555 in 2011). The Hockey FIT program sessions were delivered at GoodLife Fitness Centres Inc., Budweiser Gardens (home arena for the London Knights), or the RBC Centre (home arena for the Sarnia Sting). All measurement sessions were conducted at either Western University (London site) or the RBC Centre (Sarnia site).

#### Sample size

A recruitment target of 80 male hockey fans of the London Knights and Sarnia Sting (40 per team; 20 men per arm within each team) was based on a sample size calculation for a subsequent full-scale RCT, following methods by FFIT [15]. To detect a 5 % difference in weight loss between intervention and comparison groups at 12 months, with a standard deviation of 19.9 %, 80 % power and a 0.05 two-sided significance level, 250 men are required in each arm [50]. To allow for 25 % loss to follow-up, a very conservative estimate, the sample size was increased to 335 men in each arm. A target of 670 men reflects the need to recruit 40 male hockey fans from 17 different OHL teams. The need to recruit 40 male hockey fans per site is reflected in this pilot trial.

#### Recruitment & participants

Participants were recruited through a variety of methods: team email blasts, team social media accounts (i.e., team twitter feed posts), posters, traditional media-based advertisements (newspaper, TV, radio, community magazines), Hockey FIT website [51], word of mouth, and/or direct contact with potential recruits at the OHL team arena on game day. Those interested in participating in the study contacted the Hockey FIT Research Coordinator by email or telephone. The Research Coordinator pre-screened individuals for eligibility by phone or email; specifically, interested individuals were asked about their gender, age, height, weight and availability to attend Hockey FIT sessions on a certain day of the week, for 12 weeks, starting on a set date. Individuals deemed likely to be eligible were then invited to attend an in-person screening session. *Inclusion criteria:* Men aged 35–65 years, with an objectively measured BMI of at least 28 kg/m^2^, and meet safety requirements using the Physical Activity Readiness Questionnaire (PAR-Q) [52]. *Exclusion criteria:* Unable to comprehend the letter of information and consent documentation.

#### Screening

The formal screening process for each man lasted approximately 20 min. Screening only proceeded once participants provided written informed consent to participate in the study. Following this, men self-completed the PAR-Q; if a participant answered, “yes” to one or more of seven questions on the PAR-Q, a research team member completed a Health Care Provider Clearance form to highlight specific concerns regarding the man’s ability to perform physical activity. This form was then provided to the Study Physician and Principal Investigator who determined whether the individual was cleared to participate in Hockey FIT. To conclude the screening session, participants were taken individually to a private area for measurements of height (Seca 213 portable stadiometer) and weight (Tanita HD351 digital weight scale). All eligible men were then invited to continue with a baseline measurement session.

#### Randomization, allocation & blinding

Following baseline measurement (including seven days of tracking steps via a pedometer), participants returned for an enrolment and allocation session. The randomization sequence was computer-generated [1:1 in block sizes of 4 and stratified by site (London or Sarnia), using SAS version 9.4 (SAS Statistical Analysis Software)] and concealed using opaque envelopes until interventions were assigned (by an individual not involved in generating the randomization sequence). Participants were allocated in correspondence to the numerical order of the randomization envelopes and their order of appointments.

All participants (intervention and comparator) were provided with copies of Canada’s Food Guide [48] and the Canadian Physical Activity Guidelines [49]. All participants were also given an adverse events log and the research team member allocating participants stressed the importance of keeping the research team and/or their Hockey FIT coach informed of any injuries, illnesses, or changes in medication for the entirety of the study. Participants allocated to the intervention group were informed to keep their pedometer for use over the duration of the program. Participants allocated to the comparator group were asked to return their pedometer and continue with usual activities, without further intervention from the research team. Following completion of the 12-week measurement sessions, participants in the comparator group were invited to take part in the Hockey FIT program, following the exact same protocol as the intervention group.

Due to the nature of the study, participants and coaches could not be blinded to group allocation. During 12-week measurement sessions, a blinded assessor conducted all weight measurements in a separate room; no other measurements were blinded.

#### Hockey FIT program

##### 12-week active phase

The Hockey FIT program uses group-based delivery to help men achieve changes in health behaviours and indicators. The program was delivered by two trained Hockey FIT coaches (one Head Coach and one Assistant Coach) to groups of 20 men, over 12, weekly, 90-min sessions. See Table 1 for an overview of the 12-week program.

**Table 1:** Overview of the Hockey fans in training program (12-week active phase)

Classroom education component	Exercise component
Week 1: Introduction to Hockey FIT & Physical Activity Prescription	
70 min: • Introductions, program overview, ground rules • Discuss eating habits, exercise and activity levels • Introduction to lifestyle prescriptions (Rx), goal-setting, and tracking • Set Physical Activity Rx	20 min: • Group walk
Week 2: Healthy Eating Overview & Healthy Eating Prescription	
65 min: • Review healthy living goals and tracking forms • Set new Physical Activity Rx • Food groups & eating a healthy diet • Formal introduction to SMART goal setting • Set Healthy Eating Rx	25 min: • Group walk
Week 3: Meal Planning & Weight Loss	
60 min: • Review healthy living goals • Set new Physical Activity Rx • Avoiding compensation/trade-off behaviour • Healthy eating planning • Health benefits of losing weight • Calculating 5–10 % weight loss • Importance of support from others	30 min: • Introduction to principles of fitness (10 min) • Warm up exercises (8 min) • Flexibility exercises (2 min) • Walk (10 min)
Week 4: Becoming Fit & Exercise Prescription	
65 min: • Review healthy living goals • Set new Physical Activity Rx • Health benefits of exercise • Overcoming barriers to exercise • Heart rate and Rating of Perceived Exertion • Simple fitness test (STEP test) • Local amenities • Set Exercise Rx	25 min: • Warm up (5 min) • Aerobic exercise (15 min) • Cool down (5 min)
Week 5: Alcohol & Weight Gain	
50 min: • Review healthy living goals • Set new Physical Activity Rx • Alcohol and weight gain • Alcohol units • Planning your drinking • Cutting down on sugary drinks	40 min: • Warm up (5 min) • Aerobic exercise (30 min) • Cool down (5 min)
Week 6: Stages of Change	
45 min: • Review healthy living goals • Set new Physical Activity Rx • Stages of change • Setbacks and strategies for dealing with them • Shared experiences • Weight taken (to review progress at Week 7)	45 min: • Warm up (5 min) • Strength/muscular endurance exercise (with principles of strength training using body weight) (35 min) • Cool down (5 min)
Week 7: Weight Loss	
45 min: • Review healthy living goals • Set new Physical Activity Rx • Understanding food labels and healthier foods • Hints for better eating including eating breakfast	45 min: • Warm up (5 min) • Strength/muscular endurance exercise (15 min) • Aerobic exercise (15 min) • Cool down (5 min) • Flexibility exercises (5 min)
Week 9: Eating Out	
45 min: • Review healthy living goals • Set new Physical Activity Rx • Making favourite meals healthier • Eating out sensibly • Nutrition in common fast foods & suggestions for making takeout meals ‘less’ unhealthy	45 min: • Warm up (5 min) • Strength/muscular endurance exercise (15 min) • Aerobic exercise (15 min) • Cool down (5 min) • Flexibility exercises (5 min)
Week 10: Avoiding Setbacks & New Exercise Prescription	
55 min: • Review healthy living goals • Set new Physical Activity Rx • Exploring myths about healthy living • Triggers for setbacks and how to avoid them • Re-do simple fitness test (Step Test) & set new Exercise Rx	35 min: • Warm up (5 min) • Strength/muscular endurance exercise (10 min) • Aerobic exercise (10 min) • Cool down (5 min) • Flexibility exercises (5 min)
Week 11: Energy Balance & New Healthy Eating Prescription	
45 min: • Review healthy living goals • Set new Physical Activity Rx • Planning your eating • The energy balance • Locus of control revisited • Set new Healthy Eating Rx	45 min: • Warm up (5 min) • Strength/muscular endurance exercise (25 min) • Cool down (5 min) • Flexibility exercises (10 min)
Week 12: Celebrating Achievements & Next Steps	
60 min: • Celebrating achievement! • Review healthy living goals & set new Rx if necessary (Physical Activity, Healthy Eating & Exercise) • Next steps – includes introduction to suite of eHealth technology support options • Program feedback • Wrap-up	30 min: • Warm up (5 min) • Strength/muscular endurance exercise (20 min) • Cool down (5 min)

All participants received Hockey FIT handbooks at the first session. Nine sessions took place at a local GoodLife Fitness club, whereas three sessions (#1, #5, #11) were held at the affiliated hockey team’s arena. It was not possible to hold all Hockey FIT sessions at the hockey team arena due to logistical reasons; however, sessions at the arenas were critical in that they provided an association between the team and program. Hockey FIT head coaches were male graduate students in Kinesiology with extensive backgrounds in coaching (one with extensive background in hockey coaching). The Hockey FIT assistant coaches were senior female university students in Kinesiology, who also had a background in playing hockey.

Sessions are comprised of: 1) classroom-based education focusing on healthy eating and physical activity, delivered in an interactive, non-didactic style; and 2) exercise sessions where men receive training in aerobic, strength and flexibility exercises, incorporating their love of hockey “off the ice” (i.e., stick handling drills with rubber pucks), and tailored to individual abilities. At the start of the program, more time is allocated to education during each session, but as the program progresses, the amount of time spent on the classroom education decreases, while the amount of time spent on group-based exercise increases. This also coincides with the intensity of the exercise sessions increasing and the educational content becoming more focused on reviewing previously learned content.

Similar to FFIT [6], the delivery of Hockey FIT, but not its core components, is flexible to: 1) allow coaches to run the program to complement their own strengths/weaknesses; 2) match the personalities of a particular group of men; and 3) work within given delivery environments (e.g., arenas, health club facilities, or community centres). Hockey FIT includes components designed to appeal to male hockey fans such as: team-based incentives (i.e., visits from players); elements of competition (i.e., during exercise sessions); and use of humour to help men address sensitive topics (i.e., weight gain).

Coaches also provide instruction on behaviour change techniques (BCTs) effective in physical activity and dietary interventions (e.g., self-monitoring, specific goal setting and review, and feedback on behaviour) [53]. These BCTs are associated with control theory [54] and were mapped to FFIT [6]; the program also draws on other BCTs including social cognitive theory and self-regulation [55]. Coaches promote peer and other forms of social support as these have been shown effective in preventing relapse [53].

At two different time points (session #4 and #10), participants completed the Step Test and Exercise Prescription (STEP) tool in order to receive a personalized target heart rate (65–85 % of estimated maximum heart rate) [41, 44]. Coaches then encouraged participants during aerobic activities to exercise at their training heart rate and/or at a rating of 5–8 on the 10-point modified Borg Rating of Perceived Exertion scale [56]. Throughout the 12 weeks, participants took part in an incremental pedometer-based walking program. Participants also set and tracked individualized lifestyle prescriptions for physical activity (steps), exercise and healthy eating; these prescriptions were modified over the 12-week program when necessary to match participant progress.

In preparing for the *Maintenance Phase*, men were introduced to eHealth technology support tools, including the Health*e*Steps smartphone app (providing a virtual coach and tools to sustain increased physical activity) and their own team’s Hockey FIT social network – a secure, closed, web-based network of their Hockey FIT group and Head Coach on Tyze Personal Networks (i.e., participants could not invite other individuals into the network).

##### 40-week maintenance phase

Participants were encouraged to continue with their lifestyle prescriptions and sustain their behaviour change with the support of the Health*e*Steps app and the Hockey FIT Social Network. Men were encouraged to use the Network to share success stories, share resources (e.g., healthy recipes), ask questions and keep in touch with others in the group, including the Hockey FIT coach. The platform was a place where resources from the program were shared and made available to all participants. In addition to being used for casual exchange, the Hockey FIT Social Network platform was also used by the Head Coach to send out standardized messages (at 1, 3, 5, 6.5, 7.5 and 8.5 months following the active phase) containing reminders and encouraging messages about sustaining healthy lifestyle behaviours.

Six months after the completion of the Active Phase, participants were invited to attend a Hockey FIT Reunion (combining participants from both sites), which consisted of a one-hour booster session followed by lunch and free entry to a London Knights vs. Sarnia Sting game (held in London, ON). The Head Coaches from both London and Sarnia ran the booster session, which focused on reinforcing topics such as goal setting, stages of change and re-setting lifestyle prescriptions, and included an exercise segment focused on aerobic activities.

#### Outcome evaluation

At each site, all participants began the study at the same time and teams of research staff completed measurement sessions over an approximate 2-week period (at each time point). All randomized men were contacted at each follow-up, including those in the intervention group who did not complete the Hockey FIT program. Questionnaires were mailed for self-completion (and a time arranged with the study team for pick-up) for any participants who agreed to assessment but were not willing or able to come to a measurement session. Since the comparator group were offered the opportunity to take part in the Hockey FIT program shortly following the 12-week measurement session, individuals in this group did not participate in any follow up measurements beyond 12 weeks.

At baseline, participant demographic and clinical characteristics were collected including: age, marital status, education, occupation, ethnicity, health status, smoking status, medical conditions/history and medications.

##### Outcome measures

The following outcome measures were taken at baseline and 12 weeks in both groups, and then again at 12 months in the intervention group only:

1. *Objectively-measured clinical characteristics:* weight (kg and % of baseline weight), BMI (calculated from weight and height in kg/m^2^), waist circumference (cm), and resting systolic and diastolic blood pressure (BP; mmHg).
2. *Self-reported physical activity and sitting time:* average steps/day, measured using Yamax Digiwalker SW-200 pedometers and self-reported by participants using a 7-day paper log [57, 58]; total physical activity (Metabolic Equivalent (MET)-minutes/week) measured using the International Physical Activity Questionnaire (IPAQ) Short Form [59]; and time spent in sedentary activity (minutes spent sitting on a typical week day) measured with the IPAQ.
3. *Self-reported eating and alcohol:* healthful eating score, measured using Starting the Conversation (STC) questionnaire [60]; fatty food, and sugary food scores, as well as fruit and vegetable consumption, measured using a modified version of the Dietary Instrument for Nutrition Education (DINE) [61] and following scoring outlined by Hunt and colleagues [15]; and total alcohol consumption (units/week), measured using a 7-day recall diary [62].
4. *Psychological and health-related quality of life:* self-esteem score, measured using the Rosenberg Self-Esteem Scale (RSES); positive and negative affect scores, measured using the International Positive and Negative Affect Schedule Short Form (I-PANAS-SF) [63]; and self-rated health, using the European Quality of Life – 5 Dimensions – 3 Levels (EQ-5D-3 L) visual analog scale (VAS) score [64, 65].

All self-reported measures were collected using paper-based, self-administered questionnaires. Outcome measures were modelled after the FFIT evaluation but included a few differences: 1) the Hockey FIT evaluation replaced the Short Form-12 with the EQ-5D-3 L questionnaire for a simple measurement of health-related quality of life and pilot of the EQ-5D-3 L for ease of use for planning for a future economic evaluation; 2) Hockey FIT added the Starting the Conversation Questionnaire to obtain a simple single score of healthful eating; and 3) Hockey FIT also included measurement of physical activity via pedometers. See Table 2 for an overview of outcome measures and measurement protocols.

**Table 2:** Overview of study outcomes and measurement details

Outcome	Equipment or instrument	Description
Objectively Measured Clinical Characteristics		
Weight (kg and % of baseline weight)	Digital weight scale (Tanita HD-351)	• Light clothing, no shoes and empty pockets • Blinded assessor post-baseline in private area
Body mass index (kg/m^2^)	Digital weight scale (Tanita HD‐351) Portable stadiometer (seca 213)	• BMI calculated using the participant’s objectively measured height and weight • Height measured without shoes
Waist circumference (cm)	Tape measure	• Follows protocol outlined by the Heart and Stroke Foundation of Canada • Two measurements taken to record an average; if measurements differ by more than 5 mm, a third measurement is taken and used in the average
Resting systolic blood pressure (BP) (mmHg)	Digital BP monitor (BP Tru BPM-100)	• Participants sit quietly for 5 min prior to the first measurement; 3 measurements will be taken, 2 min apart. The first one is discarded, and the average of the last two is recorded • Feet flat on the floor, arm free of clothing, cuff at the level of heart and arm resting, same arm used (left arm preferred), no talking
Resting diastolic BP (mmHg)		
Self-Reported Physical Activity		
Average steps/day	Pedometer (Yamax Digiwalker SW-200 with security strap)	• Participants wear pedometers during waking hours for a 7-day period (putting pedometer on upon waking and removing immediately before sleeping), but not during showering/ bathing • Participants are asked to wear the pedometer at the waist, centered over their most dominant foot • Participants record the number of steps completed each day on a paper-based tracking form
Total physical activity (MET-min/week^a^)	International Physical Activity Questionnaire (IPAQ) – Short Form	• Self-completed paper‐based questionnaire • Participants recall information on vigorous activities, moderate activities, walking, and sitting • Sedentary time is measured with a single question (minutes spent sitting on a typical week day) • The IPAQ provides guidelines for data processing and score creation
Time spent in sedentary activity (min/day)		
Self-Reported Eating and Alcohol		
Total healthful eating score	Starting the Conversation (STC)	• Self-completed paper-based questionnaire • Designed for dietary assessment and intervention in a clinical setting • Participants recall eating habits over the past few months (on average) on 8 different items • A total healthful eating score (possible range 0–16) is calculated, whereby a lower score indicates a more healthful diet
Fatty food score	Modified version of the Dietary Instrument for Nutrition Education (DINE)	• Self-completed paper-based questionnaire • Participants recall eating habits over the last 7 days • Fruit and vegetable consumption is measured with a single question • Methods published by FFIT will be followed to calculate a fatty food score (possible range 8–68) and sugary food score (possible range 3–16), with higher scores indicative of higher consumption
Sugary food score		
Sugary food score		
Total alcohol Consumption (units/week)	7-day recall diary	• Self-completed paper-based diary • Participants recall alcohol consumption (beer, cider/cooler, wine, distilled alcohol, and other) over the last 7 days • Total alcohol consumption is calculated by summing the number of drinks across the categories for each day and then summing the total number of drinks/day
Psychological and Health-Related Quality of Life		
Self-esteem score	Rosenberg Self Esteem Scale (RSES)	• Self-completed paper-based questionnaire • Consists of 10 items assessing global self- esteem; 5 items have positively worded statements and 5 are negatively worded • Participants are asked to reflect on their current feelings (i.e., the extent to which they agree or disagree with each statement) • A summary score (possible range 0‐30) is calculated to provide a measure of self-esteem, where higher scores indicate higher self- esteem
Positive Affect Score	International Positive and Negative Affect Schedule Short Form (I‐PANAS‐SF)	• Self-completed paper-based questionnaire • Consists of 10 words that describe different feelings or emotions (half positive, half negative) • Participants rate how they normally feel, from 1 = never to 5 = always • Positive and negative affect sub‐scale scores are calculated (both range 5‐25), where higher scores indicate higher levels of positive affect, and negative affect, respectively
Negative Affect Score		
Self-Rated Health [visual analog scale (VAS) score]	European Quality of Life 5 Dimensions Questionnaire - 3 Level Version (EQ‐5D- 3 L)	• Self-completed paper-based questionnaire • For purposes of this study, the VAS score will be used to assess current state of health on a scale from 0 (worst imaginable state of health) to 100 (best imaginable state of health)

^a^METs (metabolic equivalents) are multiples of the resting metabolic rate; *MET-minute* multiplying the MET score of an activity by the minutes performed

#### Participant appreciation

All participants received free promotional materials from GoodLife and Sarnia Sting/London Knights hockey teams (e.g., bags, t-shirts). Participants also received a Hockey FIT t-shirt, a Hockey FIT puck, a healthy lunch and free game tickets if they attended the reunion/booster session. Further, the intervention group received a $20 gift card to a local sports store if they attended the 12-month measurement session. Attendance raffles were held at the end of the 12-week active phase to encourage attendance at all 12 sessions (i.e., participants received one ticket for each session they attended). See Table 3 for an overview of participant appreciation.

**Table 3:** Participant appreciation in the Hockey FIT pilot study

Time point	Description
During program sessions	• GoodLife Fitness water bottles • GoodLife Fitness duffle bags • Hockey team t-shirt or track jacket
Attendance raffle	• Signed hockey jerseys • Signed hockey sticks • Cookbook • Water bottles • Gift cards • Baseball caps • Cereal • Umbrella
9-month reunion & booster session	• Hockey FIT t-shirts • Hockey FIT pucks • Gift bags from sponsor (Bank of Montreal) • Healthy lunch • Hockey game ticket (London Knights vs. Sarnia Sting)
12-month measurement session (Intervention Group)	• $20 gift cards to a local sports store

#### Statistical analysis for outcome evaluation

For all outcome measures, observed values at baseline and 12 weeks will be summarized descriptively for each group. In addition, for the Hockey FIT group only, observed values at 12 months will be summarized descriptively. For data (average steps/day), participants were required to have at least three complete days of data, in order for data to be utilized in analyses [66].

Analyses will be performed following intent-to-treat principles; thus, we will include all participants with at least valid baseline data according to the randomization scheme and regardless of compliance with the intervention and data at follow-up. We will analyze data using mixed models for repeated measurements; linear mixed models will be used for continuous outcomes and generalized linear mixed models will be used for categorical and discrete outcomes. We will follow recommendations of Fitzmaurice et al. [67] and retain the baseline outcome as part of the outcome vector and constrain the group means as equal because of randomization. For all models, we will examine differences between groups at 12 weeks and changes within groups from baseline to 12 weeks (and to 12 months for the intervention group).

Terms included in models will include time, group (treatment) × time, age and site. Time will be modeled categorically with indicator variables (with baseline as the reference category). Residuals from models will be examined and subject to assumptions checks.

Based on our past experience, we do not expect substantial missing data; nonetheless, results will be valid provided data are missing at random and no imputation of data will be required by using a mixed model analysis approach [67]. To address participant dropout, we will also compare baseline characteristics of men who dropped out versus men who were included in the analysis. Interpretation of study results will primarily be based on estimation and associated 95 % confidence intervals. Two-sided *p*-values less than 0.05 will be reported as statistically significant. Analyses will be performed using SAS version 9.4 (SAS Statistical Analysis Software).

### Aims 3–5: Feasibility of recruiting and retaining overweight or obese men, acceptability of Hockey FIT program and research procedures, and Hockey FIT program optimization

#### Process evaluation data sources

Process data will be collected through self-reports, interviews, observation, questionnaires, and focus group discussions.

##### Participant self-reports at screening and information from baseline measurement sessions

Potential participants were contacted by the research coordinator and screened via email or phone to assess initial eligibility including: gender, age, height, weight, and how they heard about the Hockey FIT program. If the potential participant was deemed “likely” to be eligible after this stage, they were invited to a formal in-person screening session. Once registered in the program, participants attended a baseline measurement session, and demographic information was gathered such as age, postal code, marital status, education, occupation, and ethnicity, overall self-rated health, and clinical characteristics (described in greater detail in the outcome measures section above). These measures were collected to compare the demographics and health of the Hockey FIT participants to the health of men in the general Canadian population.

##### 12-week non-completer telephone interviews

Non-completers were defined as participants randomized to the intervention group who attended less than six program sessions (<50 %) and did not attend any of the final six program sessions.

Non-completers were contacted after all 12 sessions were completed and were asked to complete a program exit survey over the phone. Participants were asked about why they initially joined the program, why they stopped attending the program, if their involvement in the program has changed their lifestyle habits, and if (and how) the program could be changed to encourage them to complete the program.

##### Weekly attendance records

Hockey FIT coaches took attendance at the beginning of each of the 12 weekly sessions for both sites.

##### Fidelity (Program observations and post session coach reflections)

A member of the Hockey FIT research team was trained to observe program delivery at both London and Sarnia sites. All 12 weekly sessions at both sites were observed and notes were taken during the sessions. These notes detailed the overall session flow (completion of key tasks, classroom setup, interaction among participants, timing, style and method of delivery, and key lessons learned) and were summarized by the central research team. The research team member who observed the program delivery interviewed coaches after each session. Questions were specific to the experience delivering the program components and were standardized to what went well with delivery and what could be done differently.

##### 12-week participant program questionnaire

Participants randomized to the intervention group who had attended a minimum of six Hockey FIT coaching sessions (50 %), including at least one of the sessions in the final six weeks of the intervention were asked to complete a 12-week online questionnaire. The program questionnaires, created through the Qualtrics® platform, were e-mailed to participants via an individual link immediately following the final program session. A reminder was sent to men who had not completed the questionnaire after the final session. Questions explored reasons for joining, usefulness of program components, experience with the program and coaches, and any suggestions for improvement.

##### 12-week participant focus groups

At both sites, participants randomized to the intervention group were asked during sessions 11 and 12 to sign-up for a pre-determined focus group time slot. Focus groups were audio-recorded, semi-structured in nature, and moderated by a trained qualitative interviewer at both sites. Questions explored participants’ reasons for joining the program, experience with the program and health behaviour changes, and suggestions for future program improvement. Along with the interviewer, there was also an observer who made notes about group interactions, dynamics and flow of the focus group discussion. Focus groups were an hour and a half in length and the men were provided with healthy snacks, beverages and water bottles as a token of appreciation.

##### 12-week coach interviews

The four coaches (two Head Coaches and two Assistant Coaches) participated in one-on-one interviews by a trained qualitative interviewer. Questions were semi-structured and allowed for elaboration on key components of the program such as the strengths and weaknesses of the program, program delivery, and any suggested changes to the program.

##### 12-month participant program questionnaire

During the 12-month measurement session, intervention group participants were asked to fill out a questionnaire detailing their overall experience with the program and their experience with maintaining their healthy lifestyle behaviours, including use of eHealth technology support tools, following the active phase of the program. Participants were also asked whether they would recommend the program to others.

##### 12-month participant interview

All willing intervention group participants were interviewed one-on-one with trained qualitative researchers either in-person or via the telephone. Participants were asked about their experience maintaining behaviour changes made during the program, satisfaction with support from Hockey FIT staff and coaches, effectiveness of maintenance resources (Hockey FIT Social Network, Health*e*Steps app, and reunion/ booster session), and suggestions on how to improve the program.

#### Process evaluation & program optimization

These data sources will be analyzed to measure program reach, the reasons participants stayed with or opted out of the program, the extent to which coaches delivered Hockey FIT as designed, participants’ experiences of taking part in Hockey FIT, the coaches’ experience of delivering Hockey FIT, and the participants’ experience maintaining their lifestyle changes. Table 4 provides an overview of which data sources will address each process measure. The research team will track recruitment methods and reasons for study exclusion in order to address the feasibility of recruiting overweight and obese, middle-aged male hockey fans into a program focused on physical activity, exercise and healthy eating, delivered in collaboration with their favourite OHL team by trained Hockey FIT coaches. Acceptability of randomization will be estimated from the percentage of eligible men attending baseline measurements who gave informed consent to take part in the pilot trial. The research team will use findings from the process evaluation to inform program optimization.

**Table 4:** Data sources to address process measures

Process measures	Data sources
Program Reach	Participant Self-Reports at Screening and Information from Baseline Sessions 12-Month Participant Program Questionnaire 12-Month Participant Interview
Reasons Participants Stayed With/Opted Out of the Program	12-Week Participant Focus Groups Weekly Attendance Records 12-Week Non-Completer Telephone Interviews 12-Month Participant Program Questionnaire 12 month Participant Interview
Extent to which Coaches Delivered Hockey FIT as Designed	Fidelity (Program Observations and Post Session Coach Reflections) Weekly Attendance Records 12-Week Coaches Interviews
Participants’ Experience of Taking Part in Hockey FIT	12-Week Participant Focus Groups 12-Week Participant Program Questionnaire 12-Month Participant Program Questionnaire 12-Month Participant Interview
Coaches’ Experience of Delivering Hockey FIT	12-Week Coaches Interviews Fidelity (Program Observations and Post Session Coach Reflections)
Participants’ Experience Maintaining Lifestyle Changes	12-Month Participant Program Questionnaire 12-Month Participant Interview

#### Qualitative analysis for process evaluation

Responses from the 12-week and 12-month participant program questionnaires and the 12-week non-completer telephone interviews will be read to identify key themes, and provide context to the quantitative responses, including further explanation on the main elements men liked and/or did not like about the Hockey FIT program, and suggested improvements or additions. We will ensure consistency in the data collected by looking at data from multiple sources, or by ‘triangulating’ the data sources [68]. To ensure robust and consistent analysis, we will use a research team with multiple investigators.

The audio-recorded 12-week participant focus group discussions, 12-week coach interviews, and 12-month participant interviews will be transcribed verbatim. Three members of the research team will analyze transcripts from the 12-week participant focus groups and coach interviews. The coding frame will be based on our research aims but will also allow for unanticipated themes to emerge and be systematically explored [69]. If there is any disagreement amongst the data sources, the research team will discuss in order to determine the most appropriate coding list and description.

The program observations and weekly coach reflections (program fidelity) will be included in the qualitative analysis to ensure accuracy of the analysis process and to look for confirmatory data. The 12-month participant interviews augment our qualitative analysis. We will look for both unique as well as confirmatory elements. The previously developed coding list will be used, if appropriate, and augmented to include new findings.

To ensure reliability and trustworthiness of both our analysis process and the results of our coding process, narrative summaries will be developed. These summaries will include the 12-week participant focus groups, 12-week coach interviews and 12-month participant interviews, organized primarily according to each process measure (see Table 4 for a review of process measures). The entire research team will meet to discuss and finalize the summaries.

## Discussion

In general, men suffer poorer health outcomes on most measures of health status and it is a challenge to engage men in healthy living initiatives. Hockey Fans in Training is based on a gender-sensitive approach that appeals to men, but tailored to delivery within a Canadian context. As observed by Evans et al. we “…need to move from description toward interventions in ways that mobilize what we have learned about masculinities and men’s health and illness practices. Furthermore, men-centered interventions should also be targeted to specific sub-groups, while being responsive to men’s particular life course health issues” [3].

In this study our aims are to: adapt FFIT and integrate this with Health*e*Steps^TM^ to create Hockey FIT; examine the potential for Hockey FIT to improve health in overweight/obese men; evaluate feasibility (recruitment, retention) and acceptability of Hockey FIT; and finally to optimize Hockey FIT using findings from a process evaluation. If our pilot results are promising, we intend to conduct a large-scale pRCT to be able to gain confirmatory evidence on effectiveness and cost-effectiveness of Hockey FIT, as well as acceptability of the Hockey FIT program in overweight and obese men. These future efforts will then be used to support scale-up of the program across a broad distribution of settings and communities, with the overall goal of improving men’s health.

## Abbreviations

BCTs: Behaviour change techniques
BMI: Body mass index
BP: Blood pressure
DINE: Dietary Instrument for Nutrition Education
EQ-5D-3L: European Quality of Life – 5 Dimensions – 3 Level
FFIT: Football Fans in Training
Hockey FIT: Hockey Fans in Training
I-PANAS-SF: International Positive and Negative Affect Schedule Short Form
IPAQ: International Physical Activity Questionnaire
MET: Metabolic Equivalent
OHL: Ontario Hockey League
PAR-Q: Physical Activity Readiness Questionnaire
pRCT: pragmatic Randomized Controlled Trial
RSES: Rosenberg Self-Esteem Scale
STC: Starting the Conversation
STEP: Step Test and Exercise Prescription
VAS: Visual analog scale
